# Supplementary material for: Monoterpene Indole Alkaloids with Cav3.1 T-Type Calcium Channel Inhibitory Activity from Catharanthus roseus
Source: Molecules. 2021 Oct 28;26(21):6516. doi: 10.3390/molecules26216516 (PMC8587030; doi:10.3390/molecules26216516)
Supplement: Supplementary file 1 [file molecules-26-06516-s001.zip › Supplementary Materials.pdf]

Supplementary Materials

Monoterpene Indole Alkaloids with $Ca_v3.1$ T-Type Calcium Channel Inhibitory Activity from *Catharanthus roseus*

Zhen-Tao Deng [1,2,3,†], Wen-Yan Li [2,†], Lei Wang [2], Zhi-Ping Zhou [1,2], Xing-De Wu [2,4,*], Zhong-Tao Ding [1,*] and Qin-Shi Zhao [2,*]

1 Key Laboratory of Medicinal Chemistry for Natural Resource, Ministry of Education and Yunnan Province, School of Chemical Science and Technology, Yunnan University, Kunming 650091, China; dengzhentaoa@mail.kib.ac.cn (Z.-T.D.); zhouzhiping@mail.kib.ac.cn (Z.-P.Z.)

2 State Key Laboratory of Phytochemistry and Plant Resources in West China, Kunming Institute of Botany, Chinese Academy of Sciences, Kunming 650201, China; liwenyan@mail.kib.ac.cn (W.-Y.L.); laralei@163.com (L.W.)

3 University of Chinese Academy of Sciences, Beijing 100049, China

4 Key Laboratory of Ethnic Medicine Resource Chemistry, State Ethnic Affairs Commission & Ministry of Education, Yunnan Minzu University, Kunming 650500, China

* Correspondence: wuxingde@mail.kib.ac.cn (X.-D.W.); ztding@ynu.edu.cn (Z.-T.D.); qinshizhao@mail.kib.ac.cn (Q.-S.Z.); Tel.: +86-871-65223058 (Q.-S.Z.)

† These authors contributed equally to this work.

CONTENT

**Figure S1** ^{1}H NMR spectrum (600 MHz, $CDCl_3$) of **1**
**Figure S2** ^{13}C and DEPT NMR spectrum (150 MHz, $CDCl_3$) of **1**
**Figure S3** HSQC spectrum of **1**
**Figure S4** COSY spectrum of **1**
**Figure S5** HMBC spectrum of **1**
**Figure S6** ROESY spectrum of **1**
**Figure S7** HREIMS spectrum of **1**
**Figure S8** UV spectrum of **1**
**Figure S9** IR spectrum of **1**
**Figure S10** ^{1}H NMR spectrum (600 MHz, $CDCl_3$) of **2**
**Figure S11** ^{13}C and DEPT NMR spectrum (150 MHz, $CDCl_3$) of **2**
**Figure S12** HSQC spectrum of **2**
**Figure S13** COSY spectrum of **2**
**Figure S14** HMBC spectrum of **2**
**Figure S15** ROESY spectrum of **2**
**Figure S16** HREIMS spectrum of **2**
**Figure S17** UV spectrum of **2**
**Figure S18** IR spectrum of **2**

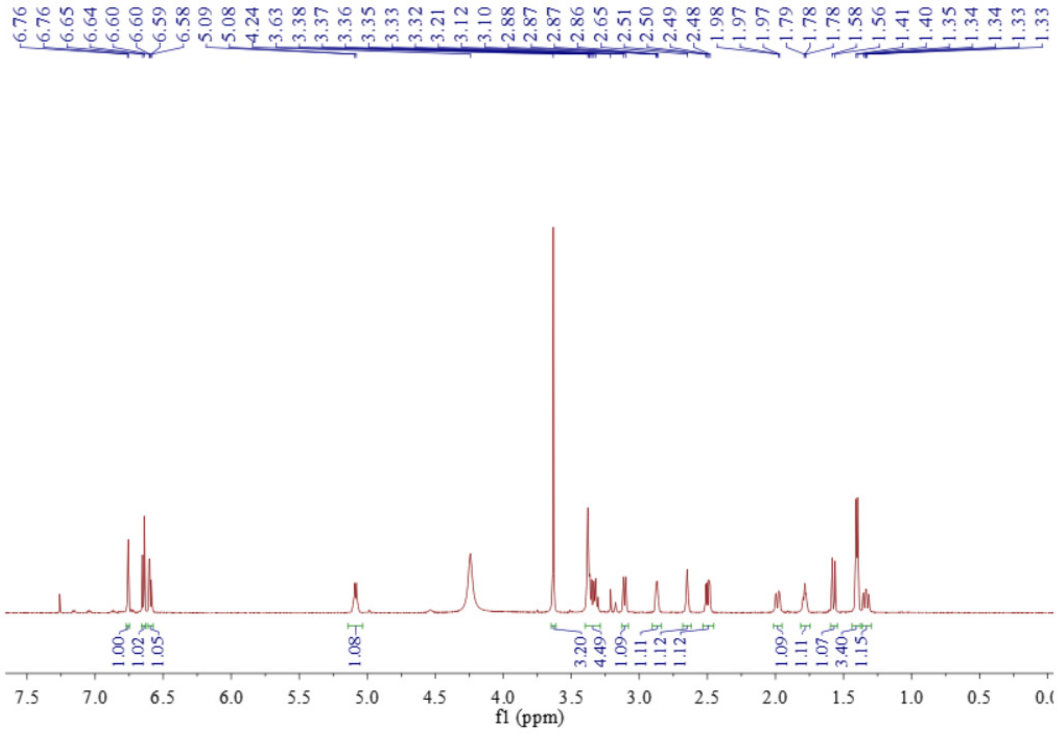

Figure S1 ^{1}H NMR spectrum (600 MHz, $CDCl_3$) of **1**

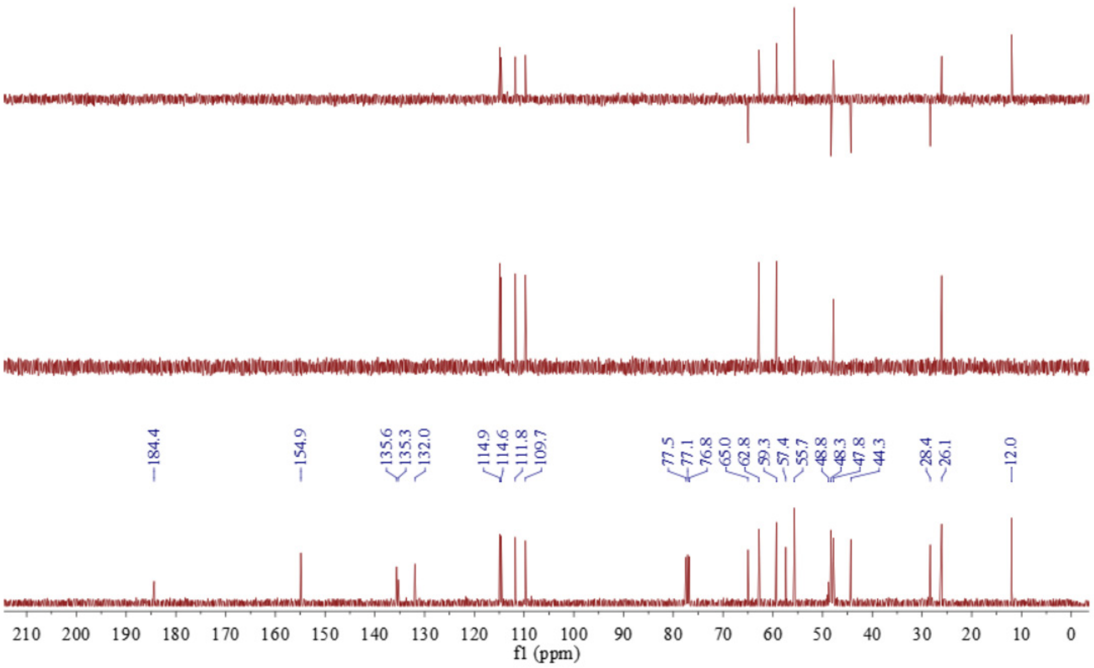

Figure S2 ^{13}C and DEPT NMR spectrum (150 MHz, $CDCl_3$) of **1**

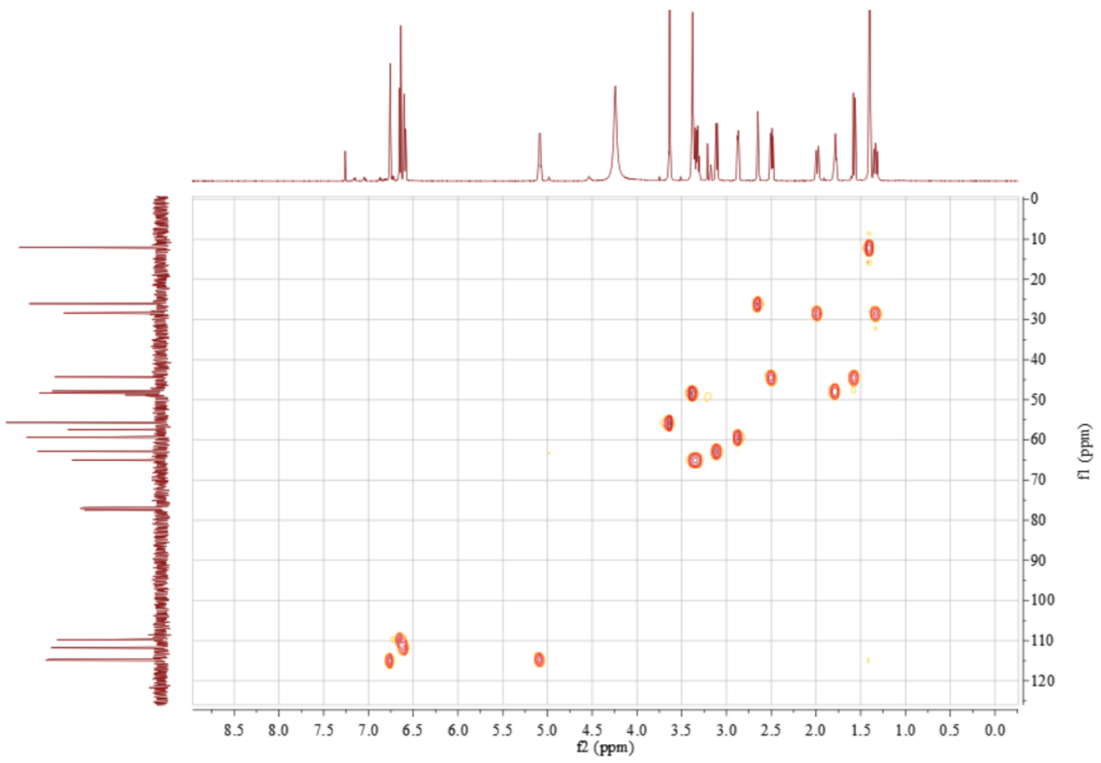

Figure S3 HSQC spectrum of **1**

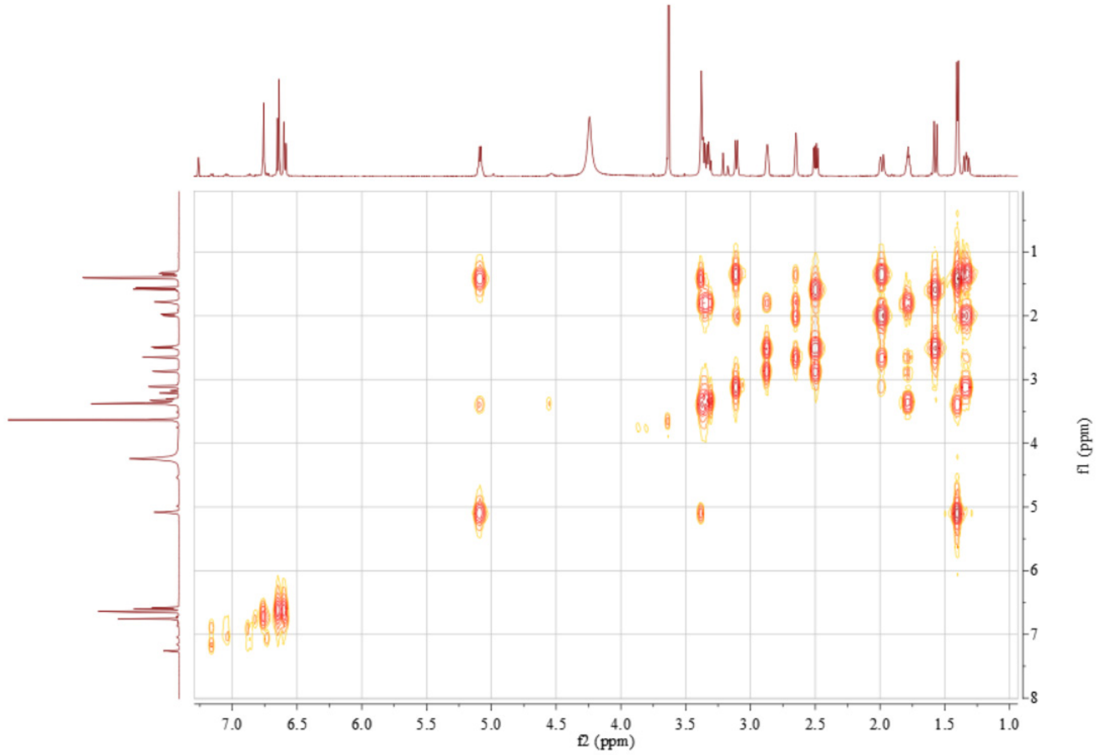

Figure S4 COSY spectrum of **1**

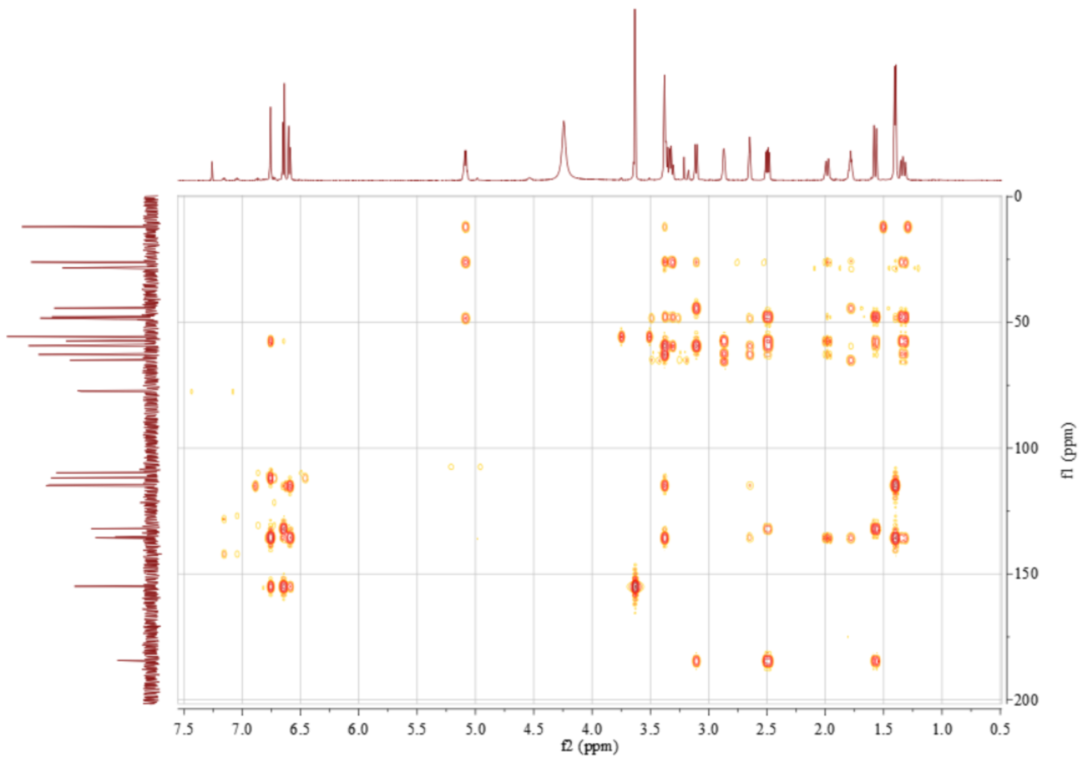

Figure S5 HMBC spectrum of **1**

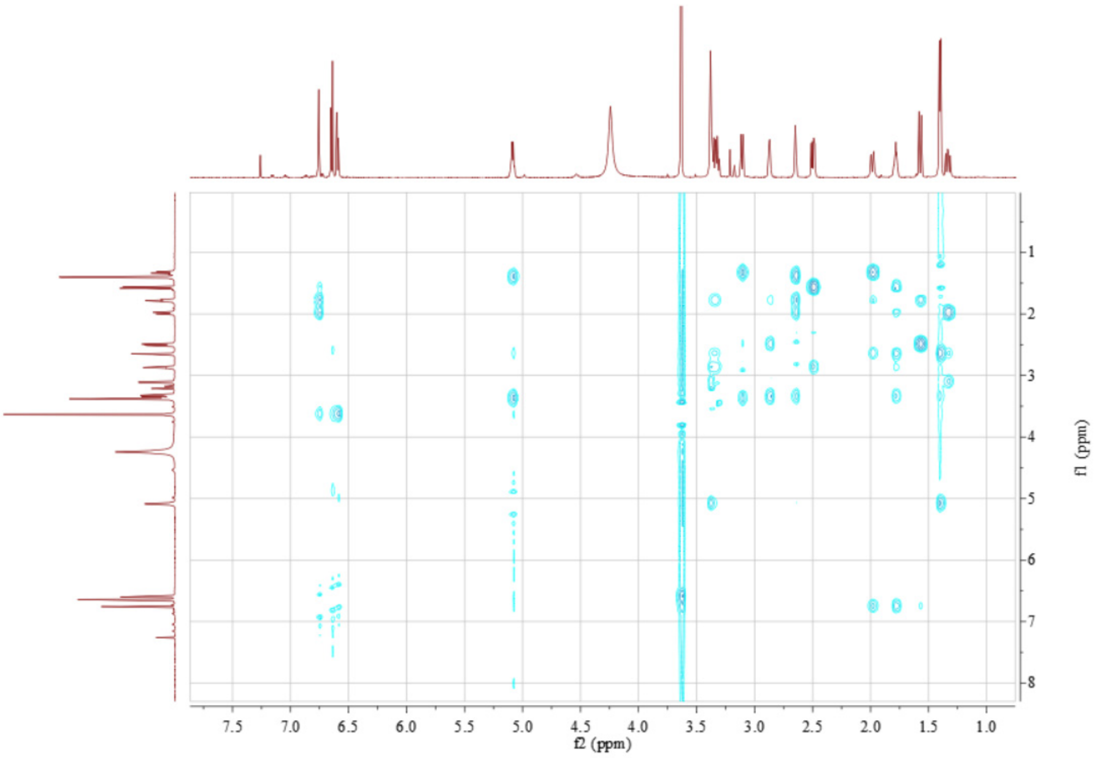

Figure S6 ROESY spectrum of **1**

Elemental Composition Report

Single Mass Analysis
Tolerance = 10.0 PPM / DBE: min = -10.0, max = 120.0
Selected filters: None

Monoisotopic Mass, Odd and Even Electron Ions
17 formula(e) evaluated with 1 results within limits (up to 51 closest results for each mass)
Elements Used:
C: 0-200 H: 0-400 N: 2-2 O: 2-4

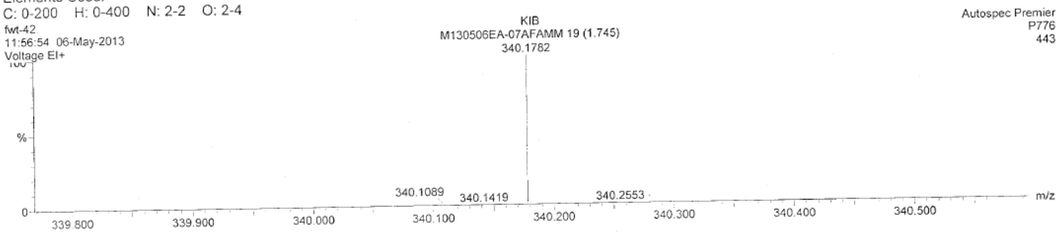

Minimum:				-10.0		
Maximum:		200.0	10.0	120.0		
Mass	Calc. Mass	mDa	PPM	DBE	i-FIT	Formula
340.1782	340.1787	-0.5	-1.5	10.0	5546234.0	C20 H24 N2 O3

Figure S7 HREIMS spectrum of **1**

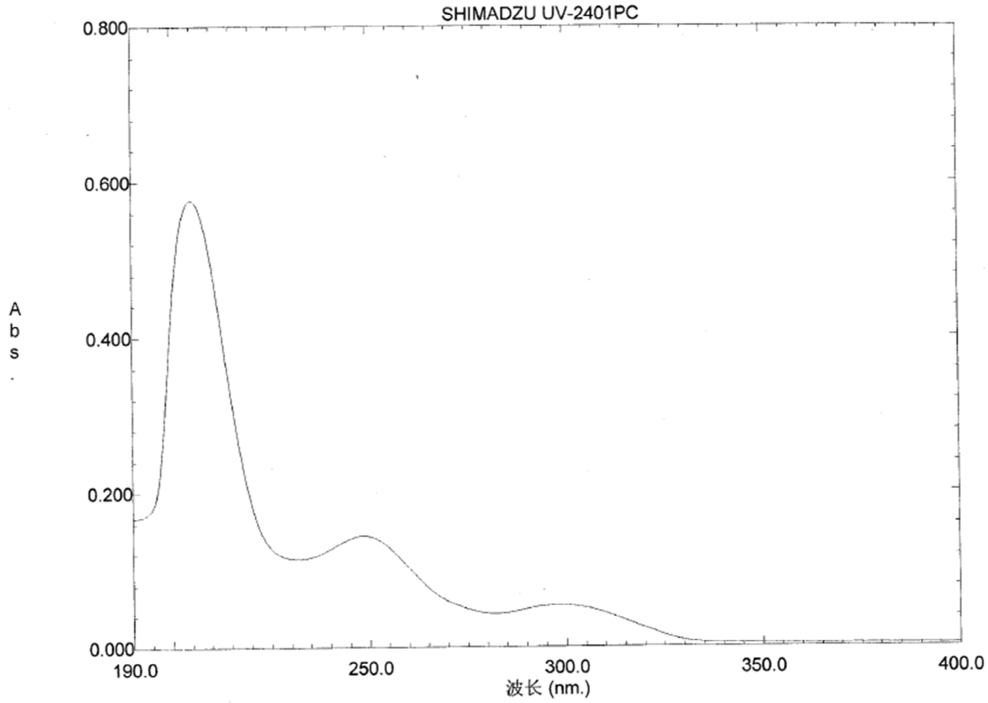

文件名: FWT-42

FWT-42 ———

样品浓度：0.0089毫克/毫升
溶剂：甲醇

创建于: 17:53 13-05-06
数据: 原始

测量模式: Abs.
扫描速度: 中速
狭缝: 5.0
采样间隔: 0.2

否.	波长 (nm.)	Abs.	
1	298.40	0.0534	3.61
2	248.40	0.1436	3.04
3	205.20	0.5766	3.64

Figure S8 UV spectrum of **1**

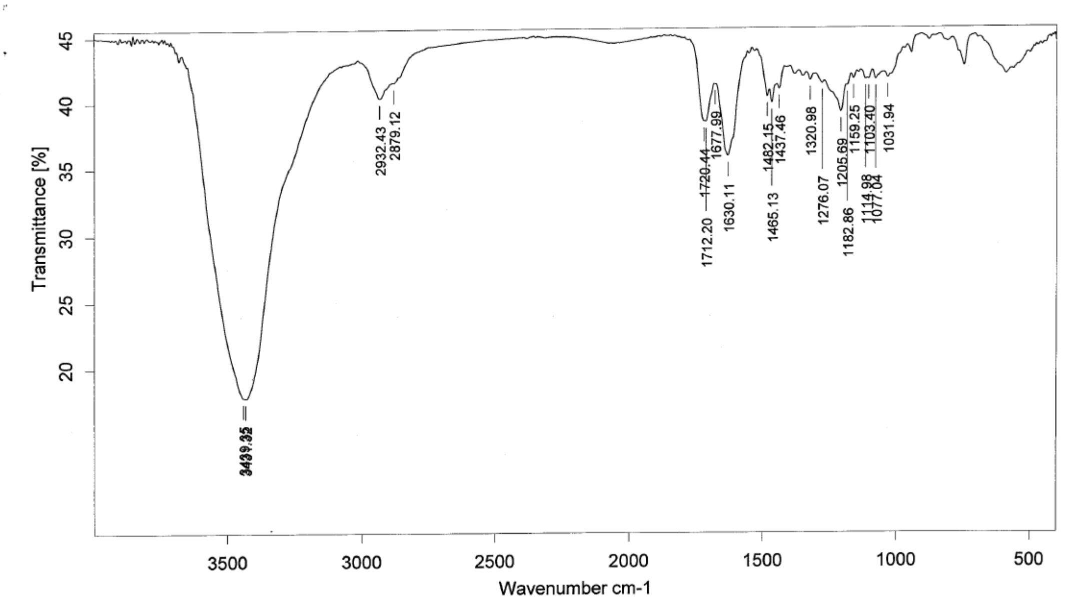

Sample : fwt-42		Frequency Range : 399.246 - 3996.32	Measured on : 08/05/2013
Technique : KBr压片	Resolution : 4	Instrument : Tensor27	Sample Scans : 16
Customer : 130508IR11	Zerofilling : 2	Acquisition : Double Sided,For	

Figure S9 IR spectrum of **1**

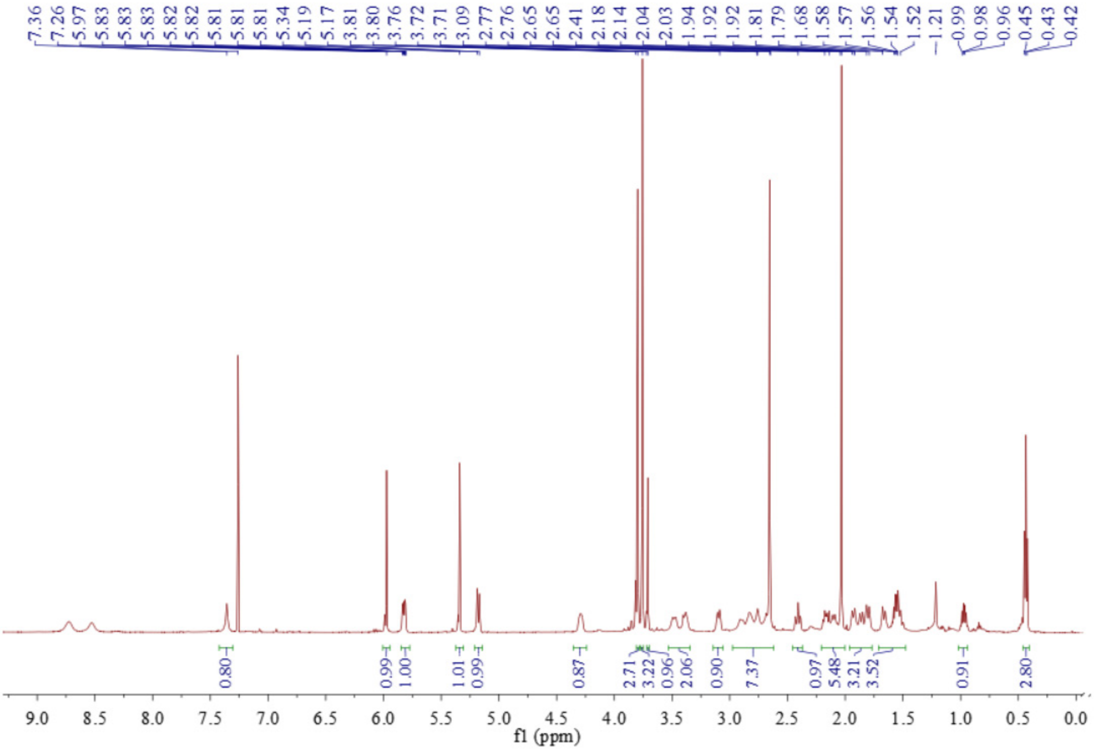

Figure S10 ^{1}H NMR spectrum (600 MHz, $CDCl_3$) of **2**

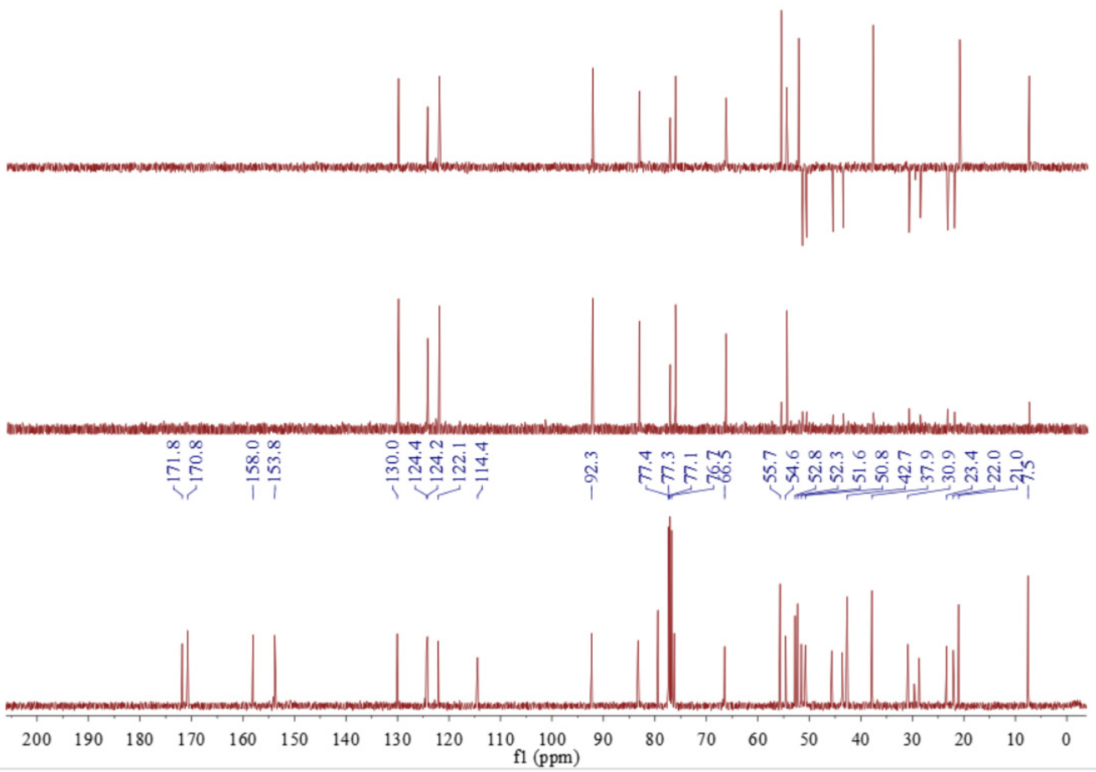

Figure S11 ^{13}C and DEPT NMR spectrum (150 MHz, $CDCl_3$) of **2**

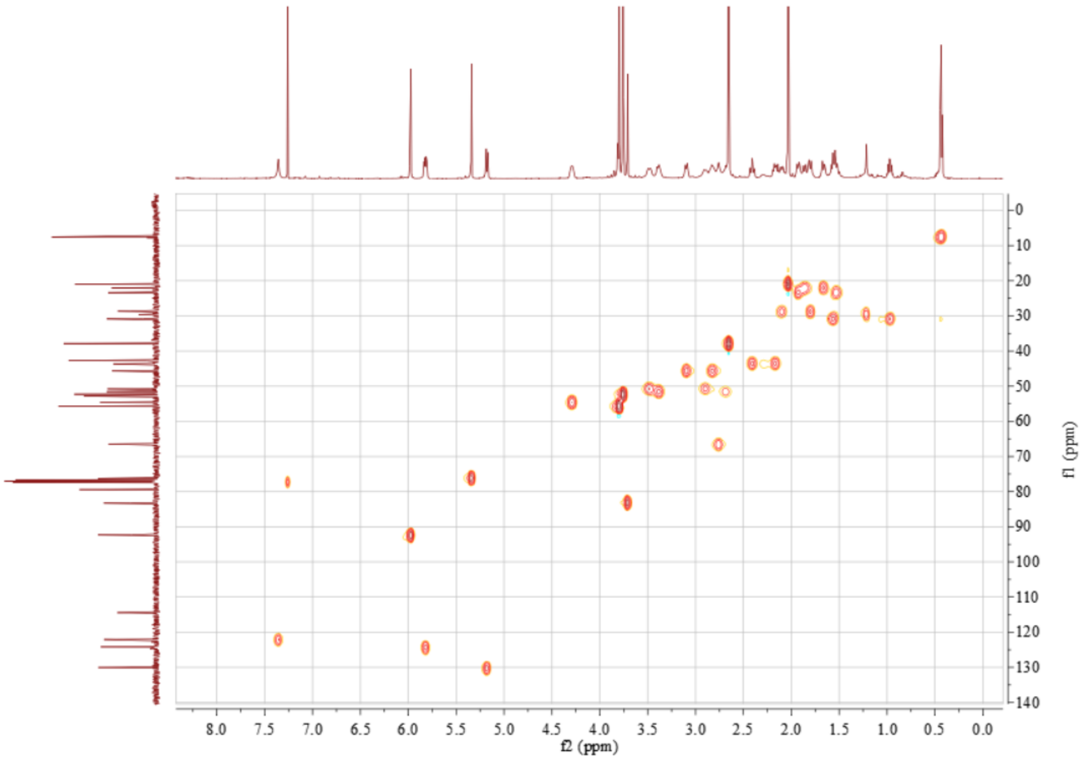

Figure S12 HSQC spectrum of **2**

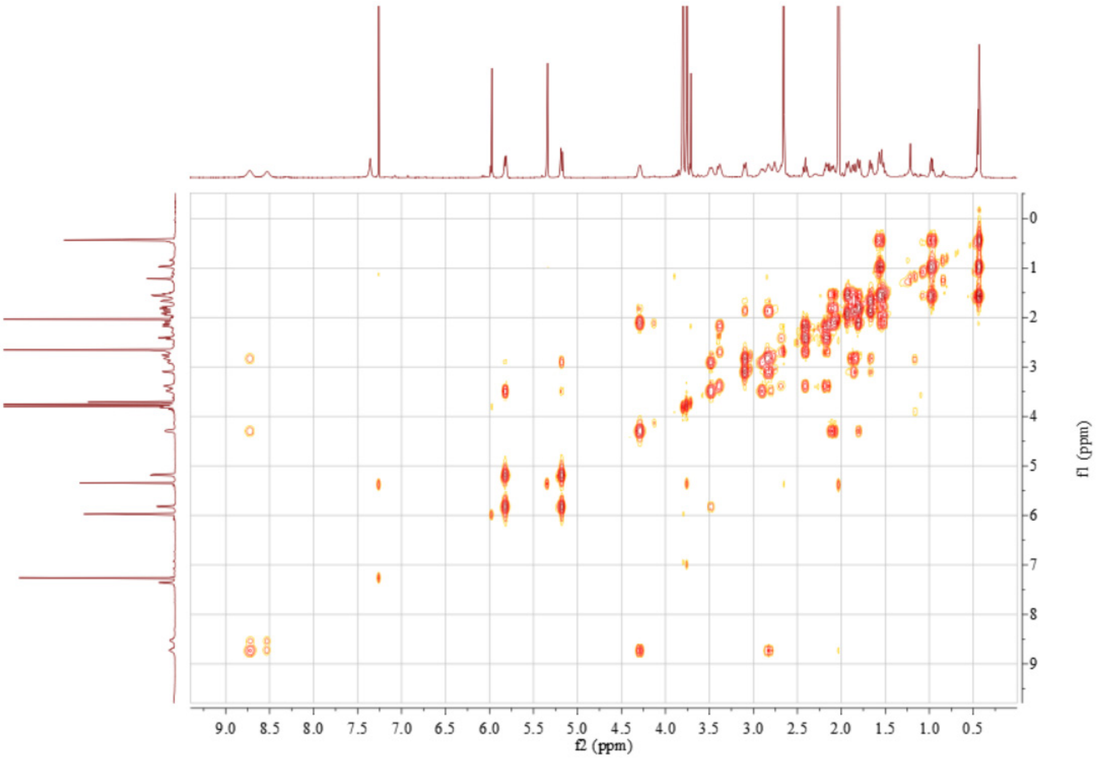

Figure S13 COSY spectrum of **2**

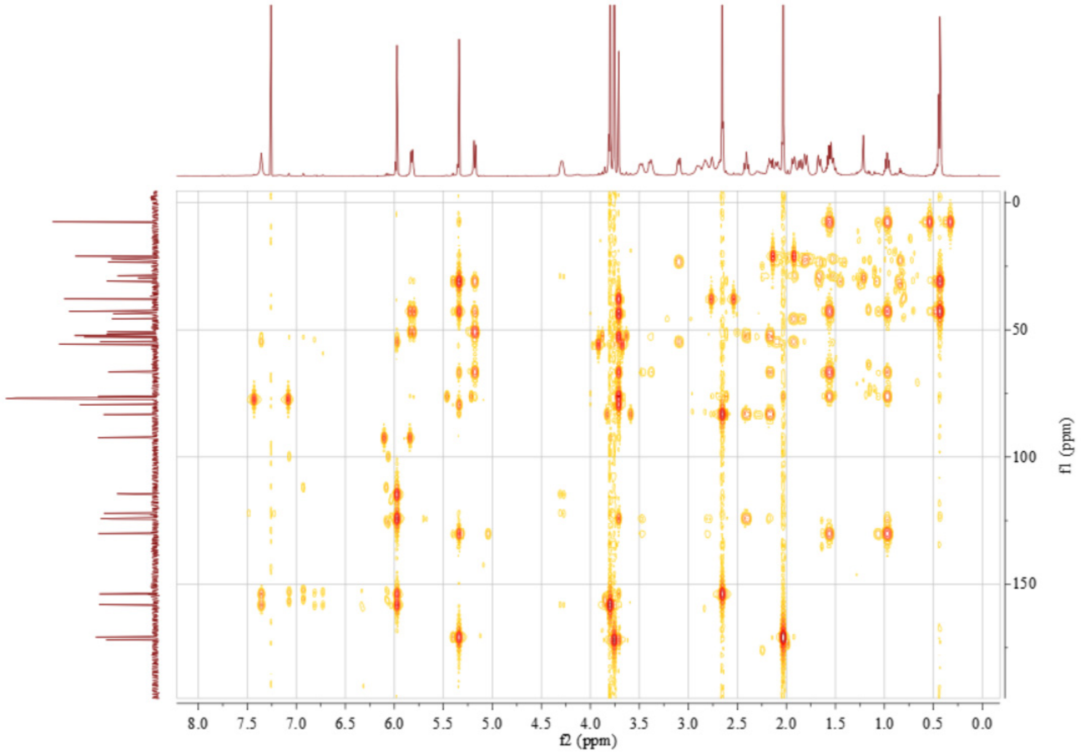

Figure S14 HMBC spectrum of **2**

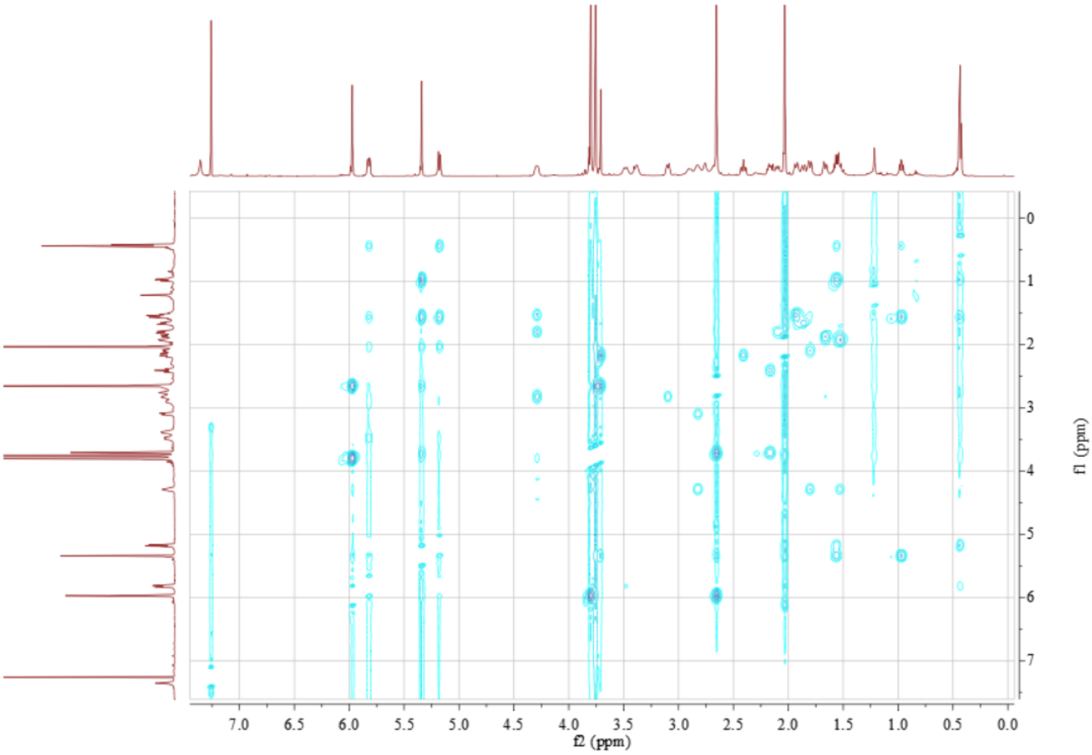

Figure S15 ROESY spectrum of **2**

Elemental Composition Report

Page 1

Single Mass Analysis
Tolerance = 10.0 PPM / DBE: min = -10.0, max = 120.0
Selected filters: None

Monoisotopic Mass, Odd and Even Electron Ions
21 formula(e) evaluated with 1 results within limits (up to 51 closest results for each mass)
Elements Used:
C: 0-200 H: 0-400 N: 3-3 O: 5-7

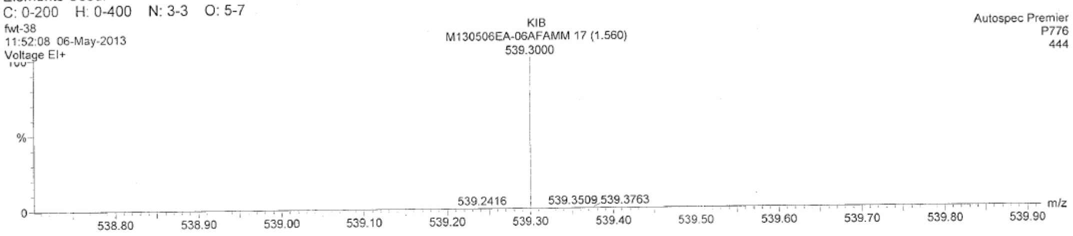

Minimum:				-10.0		
Maximum:		200.0	10.0	120.0		
Mass	Calc. Mass	mDa	PPM	DBE	i-FIT	Formula
539.3000	539.2995	0.5	0.9	12.0	5546240.0	C30 H41 N3 O6

Figure S16 HREIMS spectrum of **2**

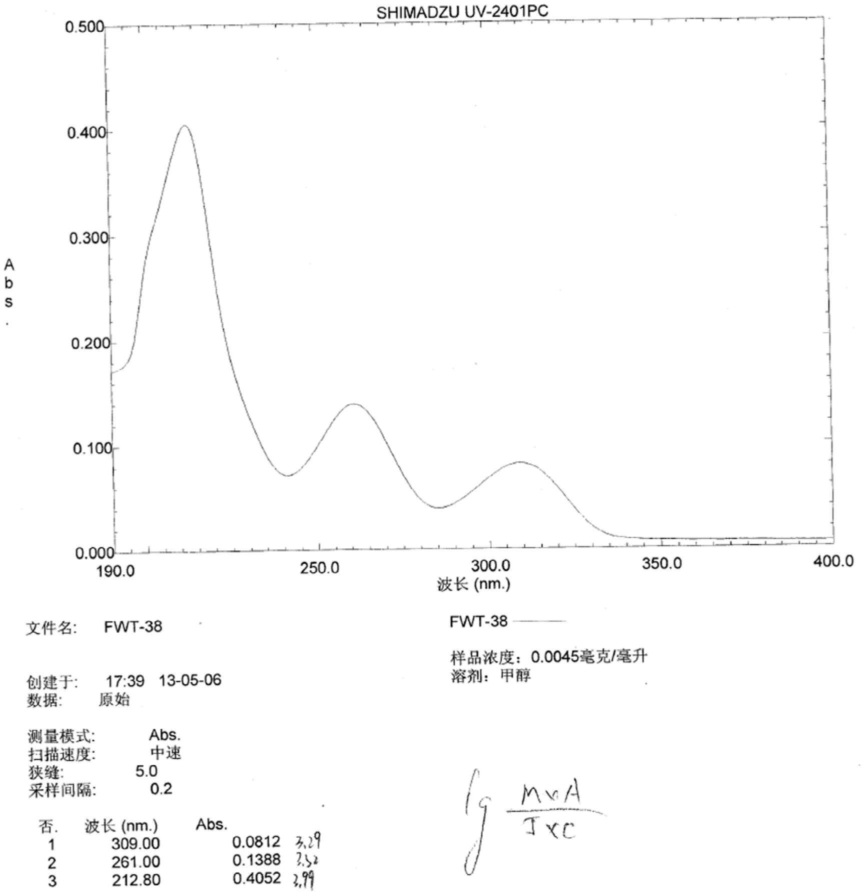

Figure S17 UV spectrum of **2**

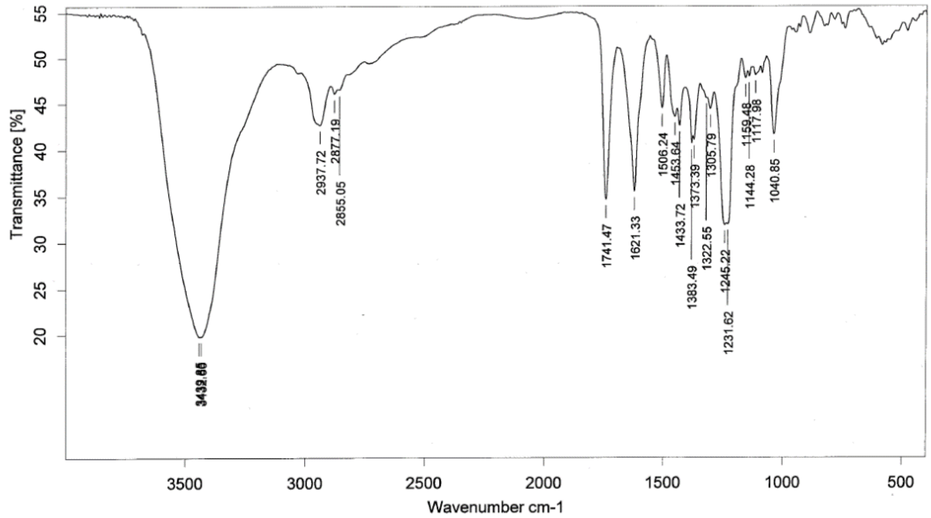

Sample : fwt-38	Frequency Range : 399.246 - 3996.32	Measured on : 08/05/2013	
Technique : KBr压片	Resolution : 4	Instrument : Tensor27	Sample Scans : 16
Customer : 130508IR10	Zerofilling : 2	Acquisition : Double Sided,For	

Figure S18 IR spectrum of **2**
